# Genetic etiology and pregnancy outcomes of abnormal fluid accumulation in fetus: A retrospective cohort study

**DOI:** 10.1371/journal.pone.0337437

**Published:** 2025-12-16

**Authors:** Meiying Cai, Na Lin, Linjuan Su, Meimei Fu, Hailong Huang, Liangpu Xu

**Affiliations:** Medical Genetic Diagnosis and Therapy Center, Fujian Maternity and Child Health Hospital College of Clinical Medicine for Obstetrics & Gynecology and Pediatrics, Fujian Medical University, Fujian Key Laboratory for Prenatal Diagnosis and Birth Defect, Fujian Clinical Research Center for Maternal-Fetal Medicine, National Key Obstetric Clinical Specialty Construction Institution of China, Fuzhou, China; Shaheed Rajaei Cardiovascular Medical and Research Center: Rajaie Cardiovascular Medical and Research Center, IRAN, ISLAMIC REPUBLIC OF

## Abstract

**Background:**

This study investigated the genetic etiology of abnormal fetal fluid accumulation, aiming to quantify pathogenic variants and correlate them with clinical outcomes to improve genetic counseling.

**Methods:**

A cohort of 305 fetuses underwent single-nucleotide polymorphism array (SNP-array) and whole-exome sequencing (WES) of amniotic fluid or cord blood.

**Results:**

Pathogenic copy number variations (CNVs) were detected in 49 cases, including aneuploidies (e.g., trisomy 21, Turner syndrome) and microdeletions/duplications. Two single-gene mutations (SNAP25, PLD1) were identified in CNV-negative cases. Non-immune hydrops (NIHF) exhibited the highest pathogenic rate (42.7%, 32/75), with non-isolated NIHF (50.0%) showing significantly higher detection than isolated NIHF (17.6%). NIHF also had the highest termination rate and postnatal abnormality rate (11%). Pleural and pericardial effusions followed in severity.

**Interpretation:**

The findings demonstrate that SNP-array and WES effectively diagnose genetic causes of fluid accumulation. While some NIHF cases may have favorable outcomes, the high termination and abnormality rates underscore its generally poor prognosis. These results emphasize the importance of comprehensive prenatal genetic testing and individualized counseling for families facing such diagnoses.

## Introduction

The genetic etiology of abnormal fetal fluid accumulation—including ascites, pleural effusion, and hydrops—remains poorly understood, with many cases lacking a clear diagnosis. Their incidence rates range from 1/15 000–1/10 000 [1,2], and prenatal diagnosis primarily depends on ultrasound [3,4]. Non-immune hydrops (NIHF), in particular, carries a high perinatal mortality risk, yet its underlying genetic causes are often unidentified [5]. While chromosomal abnormalities, monogenic disorders, and structural defects contribute to these conditions, a significant proportion of cases remain unexplained [6–8].

This study aimed to (a) determine the prevalence of pathogenic genetic variants in fetal fluid accumulation using single-nucleotide polymorphism arrays (SNP-arrays) and whole-exome sequencing (WES), (b) evaluate genotype-phenotype correlations, and (c) improve prenatal counseling strategies. By integrating genetic testing with clinical outcomes, we sought to address critical gaps in diagnosis and risk assessment for affected pregnancies.

## Methods

### Patient data

A retrospective analysis was conducted using the data of patients who visited the Prenatal Diagnosis Department of Fujian Maternal and Child Health Hospital. All patients were diagnosed with a fetal ultrasound phenotype of ascites, pleural effusion, pericardial effusion, or NIHF by experienced ultrasound physicians. An immune cause was also ruled out in all cases by maternal erythrocyte immune antibody examination. Amniocentesis was performed on pregnant women before 24 weeks of gestation, and umbilical vein puncture was performed on women >24 weeks of gestation. SNP-array was performed on the extracted fetal samples, and WES was performed on some cases with normal SNP-array. WES was performed in cases with either (1) normal SNP-array results but persistent high clinical suspicion of a genetic etiology or (2) phenotypic findings suggestive of specific monogenic disorders. The recruited cases were divided into isolated and non-isolated cases. The inclusion criteria were prenatal ultrasonographic detection of NIHF, pleural effusion, ascites, and pericardial effusion. The exclusion criteria were abnormal fetal fluid accumulation caused by immunity, infectious factors, and thalassemia gene mutations. This study was approved by the Ethics Committee of the Fujian Maternity and Child Health Hospital. All methods were performed in accordance with relevant guidelines and regulations. Written informed consent was obtained from all participants. The dataset was accessed for research purposes between 01/01/2016 and 01/07/2023. The authors did not have access to identifiable participant information during or after data collection. All data were anonymized prior to analysis.

### Ultrasonic testing

Standard prenatal ultrasound examinations were performed following International Society of Ultrasound in Obstetrics and Gynecology guidelines. When fetal effusions or skin edema were detected, comprehensive evaluation of thoracic, abdominal and pericardial cavities was conducted. Pregnancy outcomes and genetic test results were subsequently obtained. For continuing pregnancies, serial ultrasound monitoring was performed to track fetal fluid accumulation progression, with follow-up of delivery and neonatal outcomes.

### SNP-array

After fetal DNA was extracted and maternal contamination was detected, an SNP-array was performed. The CytoScan HD chip (Affymetrix) was used for DNA digestion, amplification, purification, fragmentation, labeling, hybridization with the chip, washing, scanning, and data analysis in strict accordance with the standard procedures provided by Affymetrix. The chip contains both a single-nucleotide polymorphism probe and an oligonucleotide probe. The results of the SNP-array were analyzed using CHAS software and bioinformatics tools, and chromosome microdeletions and microduplications were determined according to the scatter diagram distribution of the DNA copy number. Because of the clinical significance of copy number variations (CNVs)>100.00 kb, as documented in the literature and inpublic database analysis, the CNV comparison and analysis processes included both internal and online public databases (DGV [http://projects. The Tcag. Ca/variation], DECIPHER [http://www.sanger.ac.uk/PostGenomics/decipher], human Mendel online [http://www.omim.org], and UCSC [http://www.genome.UCSC.edu/]) as references. According to the American College of Medical Genetics and Genomics guidelines for interpreting CNV results, CNVs were classified as benign, possibly benign, pathogenic, possibly pathogenic, and variants of uncertain significance (VUS).

### WES

For WES, fetal DNA was disrupted, the library was prepared, the target gene exons and DNA near the cut region were captured and enriched using the Roche KAPA Hyper Exome chip, and mutations were detected using the MGISEQ-2000 sequencing platform. The quality control indices used for sequencing data were as follows: the average depth of sequencing in the target region was ≥ 180 × , and the proportion of sites with an average depth of >20 × in the target region was > 95%. The sequenced fragment was compared with the UCSC hg19 human reference genome to eliminate duplicates. GATK was used for base mass value correction of SNV, INDEL, and genotyping. Exon-level copy number variations were detected using Exome Depth software. Gene naming was performed according to the Human Genome Organization Committee on Gene Nomenclature. The variants were named according to the nomenclature of the Human Genome Variation Society. Annotation and screening were performed using the patients’clinical information, population and disease databases, and biological information prediction tools. The pathogenicity classification of the variations was based on the American Society for Medical Genetics and Genomics, American Society for Molecular Pathology guidelines for the interpretation of sequence variations, and the ClinGen Working Group on the Interpretation of Sequence Variation and the British Society for Clinical Genome Sciences. The mutation sites were classified as benign, possibly benign, pathogenic, and possibly pathogenic, and their clinical significance was unclear.

### Pregnancy outcome and follow-up

In cases of continued pregnancy, ultrasound reviews were conducted every 2 weeks to monitor fetal growth and development and intrauterine conditions, including changes in internal fluid accumulation. Details regarding delivery and newborn outcomes were obtained during follow up (delivery mode is determined by obstetric indications, and the neonatal treatment plan is formulated by specialists). In cases of pregnancy termination or intrauterine death/abortion, necropsy results or fetal appearance were documented. Follow-up data were acquired from hospital databases.

### Statistical analysis

All statistical analyses were performed using SPSS 21.0 (IBM Corp., Armonk, NY, USA). Categorical variables were expressed as frequencies and percentages [n (%)], with group comparisons performed using χ2 tests or Fisher’s exact tests, as appropriate. Continuous variables were assessed for normality using Shapiro-Wilk tests. Genetic associations underwent Benjamini-Hochberg FDR correction for 12 primary phenotype comparisons (q < 0.05), while clinical outcomes used nominal p < 0.05 thresholds.

## Results

### Ultrasonic phenotypes of fetuses with abnormal fluid accumulation

The age of the pregnant women in this study ranged from 18 to 46 years, and the first detection occurred from 12^+5^ to 39^+3^ weeks of pregnancy. Among the 305 cases of abnormal fluid accumulation, 19.3% (59/305) had fetal ascites (7 isolated and 52 non-isolated), 10.2% (31/305) had fetal pleural effusions (6 isolated and 25 non-isolated, including 5 cases with cardiac abnormality and 8 cases with thickened nuchal translucency), 45.9% (140/305) had fetal pericardial effusions (16 isolated and 124 non-isolated, including 99 fetuses with cardiac abnormalities), and 24.6% (75/305) had NIHF (17 isolated and 58 non-isolated, including 30 cases with thickened nuchal translucency).

### Genetic analysis of fetuses with abnormal fluid accumulation

SNP-array analysis of 305 fetuses with abnormal fluid accumulation identified 49 pathogenic CNVs, comprising: (1) 35 aneuploidies, including 18 cases of Turner syndrome (45,X; Cases 1–16, 39, 40), 11 cases of trisomy 21 (Cases 17–23, 33–38, 46), 3 cases of trisomy 18 (Cases 23–25), and 3 mosaic cases (Case 26: X trisomy; Case 27: Y monosomy; Case 47: X disomy/Y monosomy); (2) 5 large deletions, including 3 cases of 22q11.21 deletions (2.17-3.17 Mb; Cases 28, 29, 45), 1 case of 16p13.3p13.13 deletion (2.89 Mb; Case 30), and 1 case of 1q43q44 deletion (8.81 Mb; Case 48); and (3) 9 microdeletions/microduplications (0.15-6.4 Mb), including Xp22.33p11.3 duplication (42.5 Mb; Case 41), Xp22.33p11.22 duplication (52.65 Mb; Case 42), 15q11.2q13.1 duplication (6.16 Mb; Case 43), 3q29 deletion (1.72 Mb; Case 44), 11q12.1q12.3 duplication (2.85 Mb; Case 31), 9p24.3q21.13 triplication with concurrent 13q11q12.13 deletion (Case 32), 16p13.11 triplication (1.61 Mb; Case 49), and 11p14.1p12 deletion (6.4 Mb; Case 35) (Table 1). For the nine fetuses with abnormal fluid accumulation and normal SNP-array results, further WES was conducted. The results revealed that two fetuses had single gene mutations (Table 2). In summary, 51 pathogenic genomes were detected in the 305 fetuses with abnormal fluid accumulation.

**Table 1:** Pathogenic copy number variation (CNV) and clinical characteristics of cases with fetal abnormal fluid accumulation.

Case	Ultrasonic phenotype	SNP-array result	Size (Mb)	Pregnancy outcome
1	NIHF, thickened nuchal translucency	arr[hg19] (X)x1	–	TP
2	NIHF, left heart dysplasia, severe mitral stenosis, aortic stenosis	arr[hg19] (X)x1	–	TP
3	NIHF,Ventricular septal defect	arr[hg19] (X)x1	–	TP
4	NIHF,cystic hygroma, abnormal ductus venosus blood flow	arr[hg19] (X)x1	–	TP
5	NIHF, thickened nuchal translucency	arr[hg19] (X)x1	–	TP
6	NIHF,cystic hygroma, hypoplastic left heart syndrome, abnormal ductus venosus blood flow	arr[hg19] (X)x1	–	TP
7	NIHF,cystic hygroma, aortic coarctation	arr[hg19] (X)x1	–	TP
8	NIHF,cystic hygroma, aortic coarctation	arr[hg19] (X)x1	–	TP
9	NIHF, thickened nuchal translucency	arr[hg19] (X)x1	–	TP
10	NIHF,cystic hygroma, aortic coarctation, mild tricuspid regurgitation	arr[hg19] (X)x1	–	TP
11	NIHF, cystic hygroma, mild tricuspid regurgitation, nasal bone dysplasia, omphalocele, single umbilical artery	arr[hg19] (X)x1	–	TP
12	NIHF, thickened nuchal translucency,cystic hygroma, cardiac anomalies	arr[hg19] (X)x1	–	TP
13	NIHF, thickened nuchal translucency,persistent left superior vena cava, right subclavian artery vagus, abnormal ductus venosus blood flow	arr[hg19] (X)x1	–	TP
14	NIHF, thickened nuchal translucency,cystic hygroma, cardiac anomalies, omphalocele	arr[hg19] (X)x1	–	TP
15	NIHF,cystic hygroma, venous catheter missing	arr[hg19] (X)x1	–	TP
16	NIHF, thickened nuchal translucency,persistent left superior vena cava, right subclavian artery vagus, abnormal ductus venosus blood flow	arr[hg19] (X)x1	–	TP
17	NIHF, thickened nuchal translucency,Severe tricuspid regurgitation	arr[hg19] (21)x3	–	TP
18	NIHF	arr[hg19] (21)x3	–	TP
19	NIHF, thickened nuchal translucency	arr[hg19] (21)x3	–	TP
20	NIHF, thickened nuchal translucency,Nasal bone dysplasia, Strong left ventricular echo, abnormal ductus venosus blood flow	arr[hg19] (21)x3		TP
21	NIHF, thickened nuchal translucency,Strong left ventricular echo	arr[hg19] (21)x3	–	TP
22	NIHF, thickened nuchal translucency,Nasal bone dysplasia, abnormal ductus venosus blood flow, cardiac anomalies	arr[hg19] (18)x3	–	TP
23	NIHF, thickened nuchal translucency,Ventricular septal defect, Omphalocele, single umbilical artery, abnormal ductus venosus blood flow	arr[hg19] (18)x3	–	TP
24	NIHF,	arr[hg19] (18)x3	–	TP
25	NIHF, thickened nuchal translucency,	arr[hg19] (Y)x0-1		TP
26	NIHF	arr[hg19] (X)x2-3		TP
27	NIHF, thickened nuchal translucency,cystic hygroma	arr[hg1918p11.32p11.21(136228_14008582)x1		TP
28	NIHF, cardiac anomalies	arr[hg19] 22q11.21(18,631,364-21,800,471)x1		TP
29	NIHF, thickened nuchal translucency	arr[hg19] 22q11.21(18970562_21800471)x1		TP
30	NIHF, thickened nuchal translucency,cystic hygroma, Enhanced renal parenchymal echo	arr[hg19]16p13.3p13.13(7820618_10713286)x1		TP
31	NIHF, thickened nuchal translucency,	arr[hg19]11q12.1q12.3(59744692_62591428)x1		TP
32	NIHF, thickened nuchal translucency,	arr[hg19] 9p24.3q21.13(208454_77662508)x3, 13q11q12.13(19436286_26452180)x1		TP
33	Pleural effusion, thickened nuchal translucency,tricuspid regurgitation	arr[hg19] (21)x3	–	TP
34	Pleural effusion, cardiac anomalies, thickened nuchal translucency,	arr[hg19] (21)x3	–	TP
35	Pleural effusion	arr[hg19]11p14.1p12(30,211,776-36,615,043)x1	6.4	TP
36	Pericardial effusion,right subclavian artery vagus, nasal bone dysplasia, thickened nuchal translucency	arr[hg19] (21)x3	–	TP
37	Pericardial effusion,persistent left superior vena cava, right subclavian artery vagus	arr[hg19] (21)x3	–	TP
38	Pericardial effusion,cystic hygroma, nasal bone dysplasia,ventricular septal defect,left ventricular echo, severe tricuspid regurgitation	arr[hg19] (21)x3	–	TP
39	Pericardial effusion, omphalocele,	arr[hg19] (X)x1	–	TP
40	Pericardial effusion	arr[hg19] (X)x1	–	TP
41	Pericardial effusion, right dominant heart, right ventricular hypertrophy, mild tricuspid regurgitation, small for gestational age	arr[hg19]Xp22.33p11.3(168551_42659514)x1	42.5	TP
42	Pericardial effusion, left hydronephrosis, duplicate kidney, single umbilical artery	arr[hg19]Xp22.33p11.22(168,551-52,819,260)x1	52.6	TP
43	Pericardial effusion, small for gestational age, right ventricular free wall thickening	arr[hg19]15q11.2q13.1(22770422_28928730)x1	6.16	TP
44	Pericardial effusion, hepatic duct wall echo enhancement, strong echo in abdominal cavity	arr[hg19]3q29(195678475_197398204)x1	1.7	TP
45	Pericardial effusion,ventricular septal defect, aortic straddle, pulmonary stenosis	arr[hg19] 22q11.21(18,631,364-21,800,471)x1	3.1	TP
46	Sacites,strong echo in the left ventricle,thickened nuchal translucency	arr[hg19] (21)x3	–	TP
47	Ascites, ventricular septal defect, slightly thicker right ventricular wall, severe tricuspid regurgitation, slightly enhanced pulmonary valve echo	arr[hg19] (X)x1 ~ 2, (Y)x1	–	TP
48	Ascites,gastric duplication, intestinal duplication	arr[hg19]1q43q44(240411538_249224684)x1	8.8	TP
49	Ascites, echogenic bowel	arr[hg19] 16p13.11(14,900,042-16,508,123)x3	1.6	5 years old, normal

TP, termination of pregnancy; NIHF,non-immune fetal hydrops.

**Table 2:** Single gene mutations and clinical characteristics of cases with fetal abnormal fluid accumulation.

Case	Ultrasonic phenotype	Gene	Variation	Mode of inheritance	Source	Variation level	Pregnancy outcome
1	Pleural effusion, thickened nuchal translucency, strephenopodia	*SNAP25*	Chr20:10286817 NM_130811 c.593G > C, p.R198P	AD	denovo	P	TP
2	Pericardial effusion, tetralogy of fallot, Mitral and tricuspid valve disease with moderate regurgitation, Left and right ventricular wall thickening with enhanced echo	*PLD1*	Chr3:171392322 NM_002662.5 c.2197C > Tp.Q733* Chr3:171417591rs200121582 NM_002662.5 c.1171C > T,p.R391C	AR	Maternal paternal	P LP	TP
3	Pericardial effusion, Microcephaly, Intrauterine growth retardation	–	–	–	–	–	TP
4	Hydrops fetalis, Increased nuchal translucency	–	–	–	–	–	TP
5	Hydrops fetalis, Increased nuchal translucency	–	–	–	–	–	TP
6	Ascites, Gastrointestinal obstruction, Abnormality of the small intestine	–	–	–	–	–	TP
7	Hydrops fetalis, Increased nuchal translucency, Echogenic intracardiac focus	–	–	–	–	–	TP
8	Ascites, Pleural effusion, Intrauterine growth retardation, Echogenic fetal bowel	–	–	–	–	–	TP
9	Hydrops fetalis, Increased nuchal translucency	–	–	–	–	–	TP

AD, autosomal dominant; AR, autosomal recessive; P, pathogenic; LP, likely pathogenic; TP, pregnancy termination.

### Pathogenic genome rates with fetal abnormal fluid accumulation among different groups

**Fig 1:**
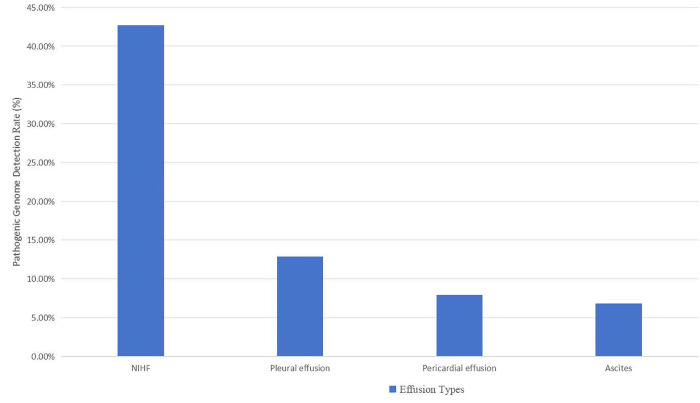
Pathogenic genome rates with fetal abnormal fluid accumulation among different groups.

The pathogenic genome rate of NIHF was the highest at 42.7% (32/75) (Fig 1), including 16 cases of Turner syndrome, 5 cases of trisomy 21 syndrome, 3 cases of trisomy 18 syndrome, 2 cases of mosaicism, 1 case of large fragment deletion, and 5 cases of microdeletion/microduplication. The pathogenic genome rate of isolated NIHF was 17.6% (3/17) and that of non-isolated NIHF was 50.0% (29/58), with a statistically significant difference between the two groups (χ2 = 5.6, P = 0.018).

The pathogenic genome rate of fetal pleural effusion was 12.9% (4/31), including 2 cases of trisomy 21 syndrome, 1 case of large fragment deletion, and 1 case of a single-gene mutation (*SNAP25* gene mutation). The pathogenic genome rate of isolated fetal pleural effusions was 16.7% (1/6) and that of non-isolated fetal pleural effusions was 12.0% (3/25). The difference between the two groups was not significant (χ2 = 1, P > 0.05).

The pathogenic genome rate of fetal pericardial effusion was 7.9% (11/140), including 3 cases of trisomy 21 syndrome, 2 cases of Turner syndrome, 2 cases of large fragment deletion, 3 cases of microdeletion, and 1 case of a single gene mutation (*PLD1* gene mutation). The pathogenic genome rate of isolated fetal pericardial effusions was 6.3% (1/16) and that of non-isolated fetal pericardial effusions was 8.1% (10/124). The difference between the two groups was not significant (χ2 = 1, P > 0.05).

The pathogenic genome rate of fetal ascites was 6.8% (4/59), including one case each of trisomy 21 syndrome, 1 case of mosaicism, 1 case of large fragment deletion, and 1 case of microdeletion. The pathogenic genome rate of isolated fetal ascites was 0% (0/7) and that of non-isolated fetal ascites was 7.7% (4/52). The difference between the two groups was not significant (χ2 = 1, P > 0.05).

### Follow-up results of fetal abnormal fluid accumulation

Of the 305 cases in this study, which had fetal abnormal fluid accumulation cases, 272 were followed, and the success rate of 89.2% (272/305). Of the cases that were followed, 47.1% (128/272) were terminated pregnancies (50 were pathogenic CNV carriers), 1.1% (3/272) were stillborn, 1.8% (5/272) resulted in perinatal death, and the remaining 50% (136/272) resulted inlive births (Table 3). Among the 136 live births examined postnatally, 11.0% (15/136) exhibited abnormal clinical phenotypes associated with effusion (Table 4), including neurodevelopmental abnormalities (n = 8), cardiac abnormalities (n = 2), ascites (n = 2), enterocyst (n = 1), pleural effusion(n = 1), and urinary system abnormalities (n = 1). The remaining 89.0% (121/136) showed no detectable abnormalities during the follow-up period.

**Table 3:** Follow-ups for thecase groups with different types of fetal abnormal fluid accumulation.

Grouping	Loss to follow-up	TP	Stillbirth	Postnatal follow-up		
				Perinatal death	Abnormal phenotype	Normal phenotype
NIHF	0 (0%)	69 (92.0%)	0 (0%)	0 (0%)	2 (33.3%)	4 (66.7%)
Ascites	10 (16.9%)	8 (16.3%)	2 (4.1%)	2 (4.1%)	6 (15.4%)	31 (79.5%)
Pericardial effusion	19 (13.6%)	41 (33.9%)	1 (0.8%)	3 (2.5%)	5 (6.3%)	71 (89.9%)
Pleural effusion	4 (12.9%)	10 (37.0%)	0 (0%)	0 (0%)	2 (11.8%)	15 (88.2%)

NIHF, non-immune fetal hydrops; TP, termination of pregnancy.

**Table 4:** Abnormal phenotypes of live infants with abnormal fluid accumulation.

Case	Prenatal ultrasound phenotype	Age	Phenotypic outcomes	Follow-up result
1	NIHF,thickened nuchal translucency,right aortic arch, left subclavian artery vagus	5 years old	Neurodevelopmental abnormalities	Developmental retardation
2	NIHF, thickened nuchal translucency	1 year and 8 months	Neurodevelopmental abnormalities	Developmental retardation
3	Ascites, echogenic bowel, enterocyst	4 years and 8 months	Enterocyst	Surgical treatment of cyst
4	Ascites, small for gestational age	1 year and 9 months	Ascites	Ascites
5	Ascites, echogenic bowel, strong echo in the left heart	9 months	Ascites	Ascites
6	Ascites, hydronephrosis	1 year and 8 months	Urinary System abnormalities	Hydronephrosis surgery
7	Ascites, left ventricle subependymal cyst	7 months	Neurodevelopmental abnormalities	Developmental retardation
8	Ascites, middle and lower abdominal bowel dilatation	1 year and 1 month	Neurodevelopmental abnormalities	Developmental retardation
9	Pericardial effusion	5 years and 6 months	Cardiac abnormalities	Cardiac surgery
10	Pericardial effusion, Tricuspid regurgitation	9 months	Neurodevelopmental abnormalities	Developmental retardation
11	Pericardial effusion,fetal growth restriction, large cardiothoracic ratio, tricuspid regurgitation	9 months	Neurodevelopmental abnormalities	Developmental retardation
12	Pericardial effusion, posterior fossa cisterna widened, left lateral ventricle widened, aortic stenosis, right heart enlargement, tricuspid regurgitation	4 years and 3 months	Cardiac abnormalities	Cardiac surgery
13	Pericardial effusion, ventricular septal defect	2 years and 10 months	Neurodevelopmental abnormalities	Developmental retardation
14	Pleural effusion	5 years and 1 month	Neurodevelopmental abnormalities	Speech and language delay
15	Pleural effusion	4 years and 8 months	Pleural effusion	Operative treatment

NIHF, non-immune fetal hydrops.

One fetus had an intrauterine ultrasound phenotype of ascites and echogenic bowel with a 16p13.11 microduplication, and the postnatal follow-up phenotype was normal. However, three fetuses with isolated effusion in the prenatal period and normal genetics then had an adverse outcome.

NIHF had the highest termination rate of 92.0% (69/75), followed by fetal pleural effusion (37.0%, 10/27) and fetal pericardial effusion (33.9%, 41/121). Among the live births with abnormal effusion, NIHF live births had the highest rate of abnormal phenotypes (33.3%, 2/6; Table 3). The normal phenotypic rates of live births with ascites, pericardial effusion, and pleural effusion were 79.5% (31/39), 89.9% (71/79), and 88.2% (15/17), respectively (Table 3).

To evaluate potential attrition bias, we compared baseline characteristics (gestational age and effusion type) between included cases (n = 272) and those lost to follow-up (n = 33) using t-tests and chi-square tests. No significant differences were observed(gestational age: t = 0.76, p = 0.45; effusion type: χ2 = 1.83, p = 0.18; all p > 0.05), supporting the assumption of missing-at-random data.

## Discussion

Chromosomal abnormalities are responsible for 29%−41% of all births with fetal pleural effusion [9]. Among these, the aneuploidy rate is approximately 12%, and when combined with other fetal abnormalities, the aneuploidy rate can reach 50%, among which trisomy 21 is common. In this study, two cases of trisomy 21 syndrome were detected in the fetal pleural effusion, consistent with the results of previous studies. However, the pathogenicity rates of isolated and non-isolated pleural effusions were not significantly different. Patients carrying *SNAP25* mutations frequently present with neurodevelopmental disorders, including intellectual impairment, motor retardation, and epilepsy, as reported in prior studies [10–13]. The *SNAP25* variant c.593G > C (p.R198P), identified here in a fetal case with pleural effusion, lies within the SNARE domain, a region critical for synaptic vesicle fusion [14]. While this variant’s association with neurodevelopmental phenotypes, our study reveals one novel observation, pleural effusion as a previously unreported feature, expanding the clinical spectrum of *SNAP25*-related disorders. Further studies are needed to validate this hypothesis.

The mechanism by which genetic abnormalities lead to fetal pericardial effusion is unclear. In this study, the pathogenic genome detection rate was 7.9% (11/140) and included three cases of trisomy 21 syndrome, two cases of Turner syndrome, two cases of large fragment deletion, three cases of microdeletion and microduplication, and one case of a single-gene mutation (*PLD1* gene mutation). Mutations in *PLD1* are known to cause developmental heart valve defect type 1 (DHVD1), characterized by congenital valve malformations such as aortic/pulmonary stenosis and regurgitation [15–18]. However, our study identifies a broader phenotypic spectrum in fetuses carrying *PLD1* mutations, including pericardial effusion – a feature not previously linked to *PLD1* dysfunction. Mechanistically, this may arise from impaired phospholipase D1-mediated signaling in pericardial mesothelial cells, which regulate fluid homeostasis via other pathways [19]. This finding implies that *PLD1* loss-of-function impacts valvulogenesis with pericardial effusion representing a novel clinical manifestation. Early prenatal detection of these features could refine prognostic assessments for *PLD1*-related disorders.

We acknowledge that our WES analysis was limited to a relatively small subset of cases (n = 9 CNV-negative samples), which may affect the generalizability of our single-gene mutation findings. The detection of pathogenic variants in SNAP25 and PLD1, while biologically plausible, requires cautious interpretation given this limited sample size. Future studies with larger WES cohorts are needed to validate these preliminary genetic associations and better characterize the spectrum of monogenic causes in fetal fluid accumulation disorders. Such efforts would benefit from multi-center collaborations to achieve adequate statistical power.

The low pathogenic genome detection rate in fetal ascites—with no chromosomal abnormalities identified in cases of isolated ascites in this study—may reflect several factors. First, technical limitations in prenatal genetic testing (e.g., resolution of conventional karyotyping or targeted CNV analysis) could miss subtle variants, particularly in non-coding regions or mosaicism. Second, isolated ascites may often arise from non-genetic etiologies (e.g., transient lymphatic dysfunction, infections, or hemodynamic imbalances), as supported by prior studies where chromosomal anomalies were rare unless accompanied by other structural defects [20]. Notably, previous reports of chromosomal abnormalities in fetal ascites primarily involved aneuploidies (trisomy 21, 18, or Turner syndrome), typically with concurrent ultrasound anomalies. Our study extends this observation: while no aneuploidies were detected in isolated ascites, we identified a novel 16p13.11 microduplication in one fetus—a finding not previously linked to fetal ascites but strongly associated with neurodevelopmental disorders (autism, cognitive impairment) [21–23]. This suggests that: The 16p13.11 variant likely represents an incidental finding rather than a causative factor for ascites, reinforcing the multifactorial nature of fetal fluid accumulation. Our cohort’ s uniqueness lies in its granular genetic profiling, which uncovered variants beyond classic aneuploidies, albeit without direct phenotypic correlation. Compared to literature emphasizing aneuploidies [24], our data imply that isolated ascites may less frequently stem from gross chromosomal abnormalities, warranting exploration of alternative mechanisms (e.g., exome sequencing for subtle mutations or non-genetic pathways).

Sturm et al. [25] showed that approximately 50% of NIHF cases are caused by chromosome aneuploidy, with Turner syndrome and trisomy 21 having the highest incidences, followed by trisomy 18, 13, and others. In this study, 26 of 76 patients with NIHF had chromosomal aneuploidy, and Turner syndrome had the highest incidence (16 cases). The pathogenicity rate of isolated NIHF differed significantly from that of non-isolated NIHF. Abnormal CNV can lead to NIHF. Su et al. [24,26] reported that the rate of pathogenic CNV was 8.3% in patients with NIHF. Abnormal CNV was also detected in five of the 76 NIHF cases in this study. In cases with no abnormalities in chromosomes or CNVs, single-gene diseases, such as metabolic diseases and RAS pathway diseases, should also be excluded [27].

Ishii et al. [28] found that NIHF cases had a poor prognosis, with all cases involving abortion, intrauterine death, or pregnancy termination. In this study, two of the six NIHF cases showed abnormal phenotypes; the first showed a developmental delay at the age of five years and the other showed a developmental delay at the age of two years. The natural resolution rate of pleural effusion ranged from 25% to 73% [29], and the perinatal mortality rate of pleural effusion ranged from 15% to 36%. In this study, three cases of perinatal fetal death with pericardial effusion were associated with cardiac malformations, underscoring the severe prognostic implications of cardiac structural abnormalities in fetal hydrops. Among the five live births with pericardial effusion, one case with isolated pericardial effusion showed a favorable response to treatment, whereas the other four exhibited additional anomalies, including cardiac and neurodevelopmental abnormalities. Notably, long-term follow-up revealed growth retardation and neurodevelopmental delays in these cases, emphasizing the need for comprehensive postnatal monitoring in fetuses with complex effusions. The prognosis of fetal ascites remains controversial. While previous studies suggest that idiopathic fetal ascites may resolve spontaneously in live births, our findings indicate a more guarded outlook. Among six live-born infants with ascites in this study, all developed abnormal phenotypes. Two cases demonstrated persistent fluid accumulation on follow-up ultrasound at one year, two exhibited growth retardation, and the remaining two required surgical intervention. These outcomes highlight the potential for long-term morbidity in fetuses with ascites, even in the absence of immediate life-threatening complications. Given the heterogeneous genetic landscape of fetal hydrops observed in this study, we propose a tiered testing protocol for clinical implementation. First-tier testing should consist of CMA to detect CNVs, which represent a significant proportion of identifiable genetic causes. For cases with negative CMA results, second-tier testing with WES is recommended, with particular focus on genes associated with lymphatic disorders, metabolic diseases, and RASopathies. In resource-limited settings where comprehensive testing may not be feasible, we suggest an alternative approach prioritizing targeted testing for common aneuploidies (trisomy 21, 18, and 13) and Noonan syndrome before considering WES.

This study has several limitations. First, the sample size was small and there was bias. It is also possible that some fetuses with abnormal fluid accumulation were terminated without genetic examination, leading to additional bias in the study. Second, this study focused specifically on genetic factors and did not account for potential confounding by gestational age or other clinical variables. The wide gestational age range (12 + 5–39 + 3 weeks) in our cohort may represent an important source of heterogeneity that was not addressed in our current analysis. Future studies incorporating comprehensive clinical metadata will be valuable to evaluate potential interactions between genetic and gestational age factors. Third, the small size of the WES cohort (n = 9) may limit the detection of rare variants with minor allele frequencies <5%, and our findings require validation in larger, population-based studies. Cases terminated without genetic testing could introduce selection bias if their genetic profiles systematically differed from those included in the analysis. The single-center design may restrict generalizability to populations with different genetic backgrounds or healthcare practices. Future multicenter studies with larger WES cohorts, combined with functional validation of candidate variants, would help improve the robustness of genetic associations and enhance our understanding of NIHF etiology. In future studies, we will continue to collect more data, improve the genetic detection of abnormal fluid accumulation in fetuses, and conduct long-term follow-ups of live births with abnormal fluid accumulation. Fourth, the live birth cohort (n = 136) represents a selected population due to high termination rates (45.2%) among cases with severe anomalies. Consequently, the observed 11% postnatal abnormality rate likely underestimates the true risk for all fetuses with effusion, as terminated cases often involved more severe phenotypes (e.g., aneuploidies, large CNVs). These findings underscore the need for cautious interpretation of postnatal risk estimates in prenatal counseling, particularly when termination is a prevalent outcome. To improve generalizability, we propose multicenter studies across diverse populations, validating variants across ethnicities and environments while reducing bias. Prioritized variants will undergo in vitro and in vivo functional validation, with multi-omics integration to uncover regulatory mechanisms.

We encourage pregnant women carrying a fetus with abnormal fluid accumulation to use diagnostic tests before pregnancy termination, including WES and SNP-array. When a fetus with effusion is diagnosed with a pathogenic genome, genetic counseling should be provided in a timely manner, and the relationship between the fetus carrying the pathogenic genome and fetal development and prognosis should be discussed with the pregnant woman and her family. While some cases of NIHF—particularly those without severe structural anomalies—may achieve favorable outcomes, our findings underscore the generally poor prognosis, as evidenced by the high termination rate and the 11% abnormality rate among live births. These results highlight the need for careful prenatal counseling and individualized risk assessment.
